# Updating and Adapting Swiss Physical Activity Guidelines: A Journey Towards Alignment With the WHO Guidelines

**DOI:** 10.3389/ijph.2024.1607539

**Published:** 2024-06-28

**Authors:** Simon Endes, Sonja Kahlmeier, Anja Frei, Thomas Radtke, Susi Kriemler, Claudio R. Nigg

**Affiliations:** ^1^ Area Health and Ageing, Ecoplan AG—Research and Consultancy in Economics and Politics, Berne, Switzerland; ^2^ Department of Health, Swiss Distance University of Applied Sciences (FFHS), Zurich, Switzerland; ^3^ Epidemiology, Biostatistics and Prevention Institute, University of Zurich, Zurich, Switzerland; ^4^ WHO Collaborating Centre for Physical Activity and Health, Zurich, Switzerland; ^5^ Department of Health Science, Institute of Sport Science, University of Berne, Berne, Switzerland

**Keywords:** physical activity, guidelines, recommendations, stakeholder process, public health

## Introduction

Physical activity (PA) has well-documented benefits for physical and mental wellbeing [1]. Over 7% of all-cause and cardiovascular disease-related fatalities, along with up to 8% of deaths resulting from 13 major non-communicable diseases can be attributed to insufficient PA [2]. These associations underscore the pivotal role that regular PA plays in safeguarding and enhancing health across the lifespan and enhancing longevity [1].

Globally, nearly one in four adults and four out of five adolescents fail to engage in recommended levels of PA [3, 4]. While Switzerland has made progress in reducing insufficient PA, approximately one in four adults and more than about one in three young people still do not meet the recommended activity levels [5].

In 2020, the World Health Organization (WHO) released updated global guidelines on PA, recommending increased efforts to promote and facilitate PA and reducing sedentary behaviour at the population level. These guidelines serve as a pivotal component within a nation’s strategy for promoting PA [6]. The Global Action Plan on Physical Activity, as endorsed by the WHO in 2018, advocates for countries to establish and regularly revise their national guidelines.

This paper presents the journey for updating the Swiss PA guidelines from 2013, emphasizing the importance of alignment with the global WHO guidelines but also with national conditions in addressing public health challenges. This publication summarizes the methodology and steps undertaken, offers a concise overview of the Swiss guidelines, and highlights the lessons learned and obstacles encountered from Switzerland’s experience as a case study for countries pursuing similar initiatives [7].

### The Process to Update the Swiss Guidelines

The process included the following aspects, based on experiences from previous updates [8] and from Germany [9]: 1) summarize the scientific evidence underpinning the 2020-edition of the WHO guidelines to identify elements to update or add to the existing Swiss guidelines; 2) systematically analyze the existing Swiss guidelines for different target groups and develop proposals for updates 3) develop and implement a participatory process to gain consensus with the main interested groups for each target group of new guidelines 4) finalize the guidelines and update the Swiss «Core document» on health-enhancing PA.

### Comparison of the Swiss Guidelines With the WHO Guidelines

Differences between the 2013 Swiss guidelines and the updated WHO 2020 guidelines were minimal, except for a few aspects. These included a recommended range of 150 up to 300 min of moderate-intensity PA or 75 up to 150 min of vigorous-intensity activity for adults, rather than a minimum of 150 min or 75 min, respectively. Furthermore, the previous requirement for PA to occur in minimum of 10-min bouts was eliminated (refer to Supplementary Table S1). For children, a significant modification was the inclusion of recommendations for sleep duration. With a few exceptions, the WHO 2020 guidelines were adopted (see Supplementary Appendix A for the updated Swiss PA guidelines [10]), including all the before mentioned adaptations, the slogan “every move counts” and the key messages. The main adaptations with explanations were as follows (see Kahlmeier et al. [7]):

- For children and adolescents aged 5–17 years, the basic recommendation for coordination and flexibility has been maintained. This goes hand in hand with the recommendation for a variety of types of PA and exercise, especially in childhood and adolescence.
- Adults and older adults: the WHO recommendation for additional health benefits of PA above the recommended threshold of minutes of PA has not been adopted in the general recommendation for the sake of clarity and to reduce the number of recommendations. However, it was included in the additional “Good to know” explanations.
- Likewise, the WHO recommendation for older adults concerning additional varied multicomponent PA that emphasizes functional balance and strength training at moderate or greater intensity on at least 2 days a week has been incorporated into the recommendation for muscle strength in combination with balance activities.
- The PA guidelines were also enriched in the text and illustrations with concrete examples of activities for better understanding.
- The most significant deviation from the WHO 2020 guidelines concerned the recommendations for individuals with special needs.
  o On one hand, a concise version of the existing Swiss guidelines for women during and after pregnancy (with minor proposed modifications, refer to Supplementary Table S1) was incorporated alongside a comprehensive but separate existing version [11].
  o Conversely, the proposed individual guidelines targeting individuals with chronic diseases or disabilities were not adopted. The working group concluded that the list of specified chronic disease conditions did not appear to be comprehensive (e.g., COPD was omitted) and was notably diverse. Furthermore, it was acknowledged that the abilities and requirements of individuals living with various diseases or disabilities varied considerably, making it impractical to address them within a single set of guidelines. Instead, a section addressing individuals living with chronic diseases or disabilities in the guidelines for children/young people, adults, older adults, and women during/after pregnancy was included. In addition, professional associations for various chronic diseases are encouraged to develop specific guidelines tailored to their respective target audiences, following the example of the guidelines for women during and after pregnancy.

### Conclusion

The Swiss expert panel of the update of the Swiss PA guidelines closely examined the WHO guidelines to ensure that the updated Swiss PA guidelines were **in harmony with the global WHO recommendations**. This alignment aimed to facilitate international collaboration and provide consistent guidance for both Swiss citizens and international stakeholders. Nevertheless, the **unique characteristics and cultural context of Switzerland were also considered**. The adaptations of the PA examples within the guidelines were made to suit the Swiss population’s preferences, habits, and the country’s diverse geography. Furthermore, this accounts for factors such as the presence of separate ministries dedicated to sports and health promotion. Moreover, this approach enhances the sense of ownership among the institutions and interested parties, thus promoting the effective adoption and implementation of the guidelines within the Swiss population.

To ensure broad acceptance and implementation, the updated guidelines underwent a process of **consultation and stakeholder engagement**. Public health agencies, healthcare providers, fitness organizations, and community groups were all invited to provide input and feedback on the proposed guidelines.

In conclusion, the process of updating the Swiss PA guidelines to align with the 2020 WHO guidelines has been a comprehensive and collaborative effort. It involved the expertise of multidisciplinary professionals, a rigorous evidence review, adaptation to the Swiss context, and stakeholder engagement. This effort emphasizes the importance of global alignment in addressing public health challenges. The updated guidelines provide Swiss citizens with evidence-based recommendations to lead healthier, more active lives, while also contributing to the global movement to promote PA for improving public health worldwide. It is hoped that this process and its outcomes will serve as a model for other countries seeking to update their own PA guidelines in alignment with global best practices.

## Key Message

The key message surrounding the issue of updating the Swiss PA guidelines and aligning them with the WHO guidelines is clear: a consistent, evidence-based approach to promoting PA is crucial to improving public health. By aligning with global recommendations, Switzerland not only ensures that its citizens receive the most up-to-date guidance, but also contributes to global efforts to combat non-communicable diseases and improve overall wellbeing. In the Swiss context, developing national guidelines, rather than simply adopting the WHO guidelines, allows Switzerland’s historical development and specific cultural characteristics to be taken into account and ensures stakeholder identification and engagement.
