# Supplementary material for: Updating and Adapting Swiss Physical Activity Guidelines: A Journey Towards Alignment With the WHO Guidelines
Source: Int J Public Health. 2024 Jun 28;69:1607539. doi: 10.3389/ijph.2024.1607539 (PMC11239331; doi:10.3389/ijph.2024.1607539)
Supplement: Supplementary file 1 [file Table1.docx]

Updating and Adapting Swiss Physical Activity Guidelines: A Journey Towards Alignment with the WHO Guidelines

Online Supplement:

In Table 1, the results of the analysis of the previous Swiss guidelines of 2013 (Federal Office of Sport FOSPO et al., 2013; Institute for Sports Sciences, University Lausanne, 2016) versus the new WHO guidelines (World Health Organization, 2020a) together with the adoptions and adaptations made are shown.

Table 1. Summary of the previous and updated Swiss and the new WHO guidelines

| Target group & aspect | Switzerland (2013) | WHO (2020) | Switzerland (2022) | |
| --- | --- | --- | --- | --- |
| Preschool children - infants | | | |  |
| - Physical activity | Free activities several times per day | Several times a day in a variety of ways, for those not yet active at least 30 minutes in prone position throughout the day | WHO 2020 adopted | |
| - Sedentary time | Avoid long inactivity | Not be restrained for more  than 1 hour at a time | WHO 2020 adopted | |
| - Sleep | No rec. | Specific number of hours/day | Integrating general rec. for sleep w/o hours/day | |
| - Screen time | No screen time up to 2-3 years of age | No screen time, >2 years not more than 1h/day | WHO 2020 adopted | |
| Preschool children – toddlers and young children | | | |  |
| - Physical activity | At least 180 minutes per day, regardless of intensity | At least 180 minutes in a variety of types, including MVPA; 3-4 years at least 60 mins of MVPA | WHO 2020 adopted | |
| - Sleep | No rec. | Specific number of hours/day | General rec. for sleep w/o hours/day integrated | |
| - Sedentary time | Avoid long inactivity | Not be restrained for more than1 hour at a time | WHO 2020 adopted | |
| - Screen time | No screen time recommended up to 2-3 years of age | >2 years not more than 1h/day | WHO 2020 adopted, link to national rec. added | |
| Children and young people 5-17 years of age | | | |  |
| - Physical activity | At least 1h/day MVPA for older children, considerably more for younger children | At least 60 minutes/day MVPA across the week | WHO 2020 adopted | |
| - Sedentary time | Avoid long inactivity and take active breaks every 2 hours | Avoid long inactivity, esp. screen time | WHO 2020 adopted | |
| - Other aspects | Do several times/ week varied activities that strengthen bones, muscles and improve balance, coordination and cardiovascular functions | Vigorous intensity and muscle and bone strengthening PA at least 3 times/week | WHO 2020 adopted;  In addition, national rec. to improve coordination & flexibility kept | |
| Adults 18-64 years of age | | | | |
| - Physical activity | At least 150 minutes of moderate or 75 minutes of high intensity PA/week | At least 150-300 minutes of moderate or 75-150 minutes of vigorous intensity PA/weeks | WHO 2020 adopted | |
|  | At least 10-minute-bouts | No minimum bouts | WHO 2020 adopted | |
| - Strength training | As additional rec. on at least 2 times/week | At least 2 times/week | WHO 2020 adopted | |
| - Sedentary time | Interrupt long sedentary time | Limit sedentary time and replace by any form of PA | WHO 2020 adopted, sedentary behavior specified | |
| Older adults > 64 years of age | | | | |
| - Physical activity | At least 150 minutes of moderate or 75 minutes of high intensity PA/week | At least 150-300 minutes of moderate or 75-150 minutes of high intensity PA/weeks | WHO 2020 adopted | |
|  | At least 10 minute bouts | No minimum bouts | WHO 2020 adopted | |
| - Strength training | As additional rec. on at least 2 times/week | At least 2 times/week | WHO 2020 adopted | |
| - Sedentary time | Interrupt long sedentary time | Limit sedentary time and replace by any form of PA | WHO 2020 adopted, sedentary behavior specified | |
| - Other aspects | As additional rec. multicomponent balance and strength training to reduce the risks of falls | Multicomponent balance and strength training | WHO 2020 adopted, integrated into general rec | |
| Pregnant and postpartum women | | | | |
| - Physical activity | Separate rec for pregnant and postpartum women | Same rec for both groups | Existing separate Swiss rec. kept | |
|  | At least 150 minutes of moderate intensity PA | At least 150 minutes of moderate intensity PA | No changes | |
|  | Higher intensity if done before pregnancy | Higher intensity for postpartum women after 6-8 weeks or if done before pregnancy | No changes | |
| - Strength training & stretching | As additional rec on at least 2 times/week | At least 2 times/week | WHO 2020 adopted | |
| - Sedentary time | Limit sedentary time and replace by any form of PA | Interrupt long sedentary time | WHO 2020 adopted, sedentary behavior specified | |
| Children and young living with disability  Adults and older adults with chronic conditions  Adults living with disability | | | | |
|  | No rec. | Separate rec. | Not adopted, no specific rec. Remarks targeting these groups added within each age group | |

NOTES: PA=physical activity, MVPA=moderate to vigorous intensity physical activity, rec=recommendation/s, WHO 2020 adopted, adapted Swiss rec
