# Supplementary material for: Updating and Adapting Swiss Physical Activity Guidelines: A Journey Towards Alignment With the WHO Guidelines
Source: Int J Public Health. 2024 Jun 28;69:1607539. doi: 10.3389/ijph.2024.1607539 (PMC11239331; doi:10.3389/ijph.2024.1607539)
Supplement: Supplementary file 2 [file DataSheet1.docx]

Appendix A: Swiss physical activity recommendations

The group on children agreed to provide guidelines for preschool children and to adopt the proposed amendments for school aged children and young adults, following the guidelines of the WHO (see Table 1, Online Supplement). Only the exact recommended hours of sleep from the WHO guidelines were not adopted, because the evidence supporting an exact number of hours was deemed insufficient for adoption. Instead, a general guideline to ensure sufficient good quality sleep was endorsed (see Figure 1).

Figure 1. Swiss physical activity guidelines graph for preschool children (Magglingen, Switzerland, 2023)


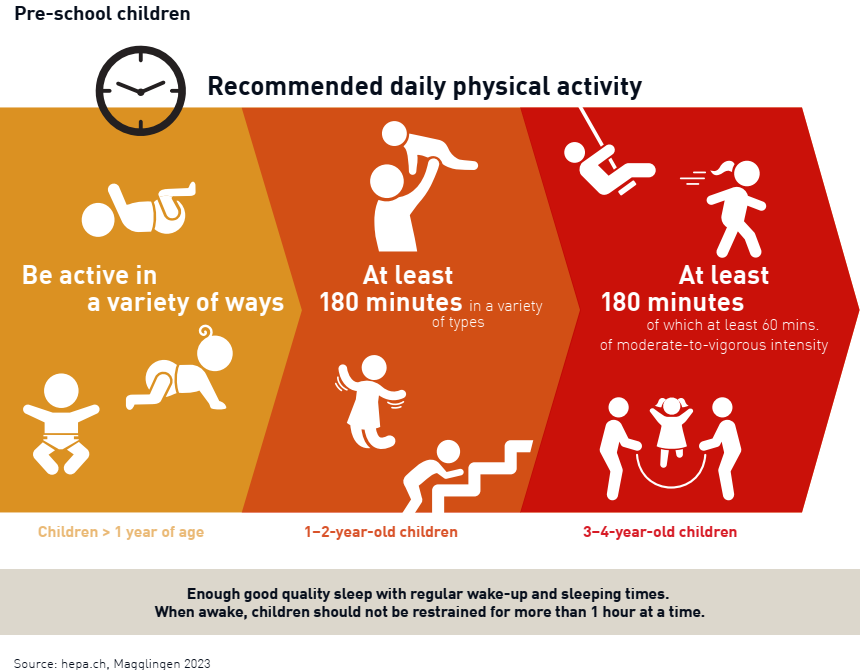


For children and young people, all proposals for amendments were approved, namely adopting an average of 60 minutes of physical activity across the week (instead of every day) and integrating reducing screen time (see Figure 2).

Figure 2. Swiss physical activity guidelines graph for children and young people 5-17 years of age (Magglingen, Switzerland, 2023)


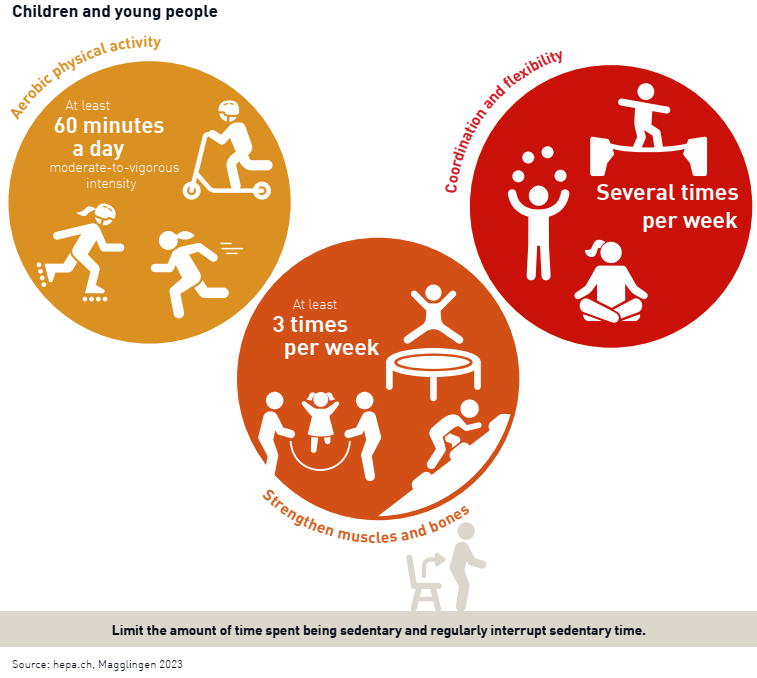


For the adults, the proposals were supported to adopt the wider range of recommended activity (up to 300 minutes of moderate intensity or 150 minutes of high intensity physical activity per week), to drop the 10-minutes bouts, to integrate strength training into the general guidelines and to adopt the amended phrasings on reducing sedentary time. Owing to the organization of the Swiss administration with separate Federal Offices of Sport and of Health, it was decided to keep the mention of both physical activity and sport in the guidelines (see Figure 3).

Figure 3. Swiss physical activity guidelines graph for adults 18-64 years of age (Magglingen, Switzerland, 2023)


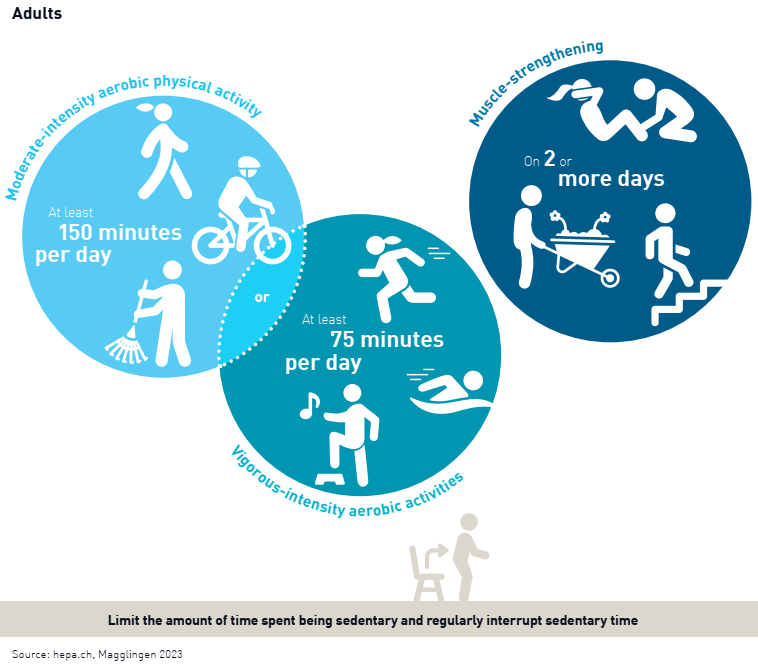


Also for older adults, all proposals were adopted, albeit some participants expressed a concern that the new upper range of recommended physical activity of up to 300 minutes could be perceived as unachievable by less fit older adults. The concurrent discussions also allowed exchange with the working group on adults during the process to make sure that changes were adopted in both groups to avoid semantic differences in the guidelines for adults and older adults (see Figure 4).

Figure 4. Swiss physical activity guidelines graph for adults > 64 years of age (Magglingen, Switzerland, 2023)


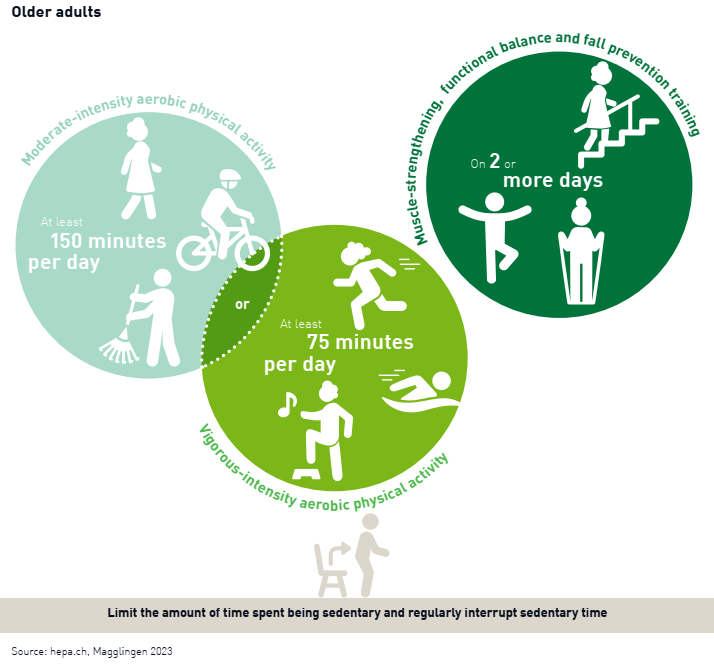


The biggest diversion from the proposed approach resulted from the working group on people with special needs. On the one hand, the working group recommended to include a short version of the guidelines for women during and after pregnancy, including the minor amendments as suggested (see Table 1, Online Supplement), but to keep the detailed existing version as a separate document (10); see Figure 5).

Figure 5. Swiss physical activity guidelines graph for women during and after pregnancy (Berne, Switzerland, 2018)

| 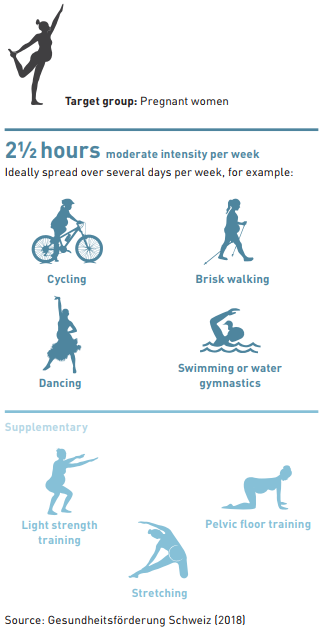 | 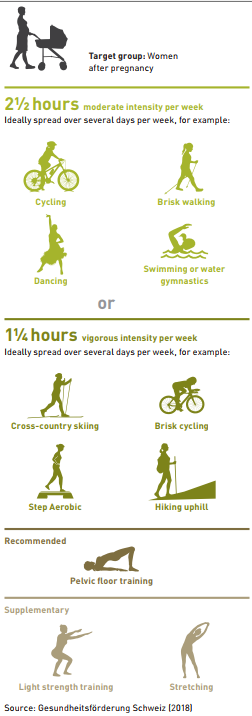 |
| --- | --- |
